# Angle-specific stiffness profiles of the achilles tendon and Triceps surae muscles: a continuous characterization

**DOI:** 10.3389/fspor.2026.1728114

**Published:** 2026-02-18

**Authors:** Yasuhiro Kunita, Naoki Ikeda, Takuya Nishioka, Shota Yamaguchi, Ayumi Yoshikawa, Daiki Hajima, Naoko Yabuno, Kengo Harato, Takayuki Inami

**Affiliations:** 1 Graduate School of Health Management (Major in Public Health, Sport and Health Sciences), Keio University, Kanagawa, Japan; 2 Institute of Physical Education, Keio University, Kanagawa, Japan; 3 Sports Medicine Research Center, Keio University, Kanagawa, Japan

**Keywords:** achilles tendon, athletes, passive ankle dorsiflexion, shear wave elastography, triceps surae

## Abstract

The stiffness of the triceps surae (TS) muscles during passive stretch has been increasingly studied using shear wave elastography (SWE). However, the continuous behavior of the Achilles tendon (AT) and its interaction with the TS muscles remain poorly understood, limiting understanding of tissue mechanics. This study aimed to characterize the angle-specific stiffness profiles of the individual TS muscles—the medial gastrocnemius (MG), lateral gastrocnemius (LG), and soleus (SOL)—and the AT during passive ankle dorsiflexion under identical experimental conditions. In this cross-sectional study, the ankles of 24 healthy university track and field athletes (age, 20.2 ± 1.3 years) were passively moved by a dynamometer from 30° of plantar flexion (PF) to 80% of maximum dorsiflexion (DF) at 1°/s (up to 25° DF). Shear wave velocity (SWV), an index of tissue stiffness, was measured at four sites (MG, LG, SOL, and AT). The percentage change in SWV from the −30° baseline was analyzed at 5° increments up to the neutral (0°) position using two-way analysis of variance. Significant SWV increases from baseline were observed at 10° PF for the MG, 5° PF for the LG, and 25° PF for the SOL. In contrast, the AT showed significant increases from 25° PF onward, with a rate of change consistently greater than that of all TS muscles across all measured angles. These findings indicate that the AT and TS exhibit asynchronous stiffening patterns: the tendon provides immediate resistance from the earliest range of motion, while the muscles remain compliant until further stretched. Thus, the tendon, rather than the muscle, primarily governs the initial resistive behavior of the muscle–tendon unit during passive stretch. Understanding this angle-specific behavior is essential for optimizing load management in rehabilitation and injury prevention.

## 1 Introduction

The mechanical properties of the muscle–tendon unit, including stiffness, compliance, and the capacity to store and return elastic energy, play a central role in athletic performance. These properties influence how efficiently force is transmitted and how effectively elastic energy is used during activities such as running and jumping (1). The triceps surae and the Achilles tendon (AT) are particularly influential in sports movements due to their anatomical continuity. During dynamic loading or ankle joint movement, contraction of the triceps surae lengthens the AT, allowing it to store and release elastic energy to enhance movement efficiency (2). However, the AT endures loads up to four times body weight during walking and up to 12.5 times during running (3, 4). During walking, elastic energy storage and return in the AT significantly contribute to the efficiency of ankle muscle–tendon work (5). Moreover, recent gait analyses highlight the dynamic interplay between medial gastrocnemius and AT length changes, underscoring the importance of considering muscle–tendon interactions when interpreting tendon loading (6). This repetitive high-stress can lead to overuse injuries, making the AT the second most common site of running-related injuries (7). Clinically, Achilles tendinopathy develops when the tendon is repeatedly exposed to loads that exceed its capacity for recovery, often in the presence of altered mechanical interaction between the triceps surae muscles and the tendon (8). Previous studies suggest that excessive or imbalanced loading across the muscle–tendon unit contributes to the development and persistence of AT disorders (9). Assessing both tissues under identical conditions in the same individuals minimizes inter-subject variability and environmental influences, enabling a clearer evaluation of how stiffness imbalances may contribute to localized tendon strain and Achilles tendinopathy. A balanced interaction of longitudinal stiffness between the muscle and tendon is therefore essential for both efficient movement and the prevention of overuse injuries (10).

Understanding the biomechanical properties of the triceps surae and AT is thus important for preventing AT disorders. The stiffness of the entire muscle–tendon unit can be derived from the passive torque–joint angle relationship (and thus the estimated muscle–tendon unit length) (11). However, this method cannot distinguish between muscle, tendon, and other soft tissues. To address this limitation, researchers have used B-mode ultrasonography to estimate the respective stiffness of the muscle and tendon from the displacement of the myotendinous junction or tendon elongation (12, 13). Yet, these approaches remain indirect, as they define stiffness based on the relationship between length change and passive torque, making it difficult to differentiate between individual muscles (e.g., gastrocnemius and soleus).

In recent years, ultrasound shear-wave elastography (SWE) has been increasingly used for the differential assessment of soft tissue stiffness. Studies using SWE have assessed stiffness during passive ankle dorsiflexion at 1–2° /s from 40° of plantar flexion (PF) (14, 15), reporting that medial gastrocnemius (MG) stiffness increases curvilinearly after approximately 18° of PF and nearly doubles by the neutral (0°) position. In many cases, stiffness begins to rise from a specific “slack angle,” where the muscle's inherent slack is taken up (15). These studies demonstrate that analyzing continuous data during dorsiflexion in 1° increments can reveal the angle-dependent behavior of stiffness.

Similarly, several studies using SWE have examined changes in shear wave velocity (or shear modulus) of the AT—parameters that reflect tissue stiffness—and have reported increases with ankle dorsiflexion (16–19). However, most of these studies measured stiffness only at specific, predetermined angles (such as the neutral 0° position) under resting conditions. In contrast, one study passively dorsiflexed the ankle at 1° /s from 50° PF and observed increased stiffness around 43° PF, indicating that the slack of the AT began to change at that point (20). However, due to limitations of their measurement device, the AT was only evaluated up to about 20° PF. Considering the results of previous studies, AT stiffness is expected to increase progressively with ankle dorsiflexion. Mechanistically, muscle tension develops gradually as cross-bridge interactions and titin-based elasticity is recruited with further stretch (21). However, the AT contains highly organized type I collagen and exhibits early uncrimping behavior, resulting in nonlinear stiffness in the initial strain range (22). These contrasting structures suggest that the tendon may stiffen earlier than the muscle when passively elongated. Nonetheless, continuous AT stiffness data up to the end of the plantar-flexed range (to the neutral 0° position) remain lacking. In the same study, Hug et al. (20) also measured the MG using the same protocol and found that its slack angle changed around 24° PF. In addition to these findings (20), previous work has demonstrated that each triceps surae muscle has a distinct slack angle. Hirata et al. (23) reported that the MG, lateral gastrocnemius (LG), and soleus (SOL) exhibit different slack angles, and that correcting for these angles significantly reduces differences in passive shear modulus among the muscles—indicating that apparent stiffness differences mainly reflect differences in muscle strain relative to slack length. More recently, Zimmer et al. (24) mapped passive and active triceps surae mechanics across ankle angles using SWE; however, they did not include the AT, leaving uncertainty about tendon–muscle relationships across the same range of motion. A recent cerebral palsy study assessed the shear modulus of the AT together with all triceps surae muscles and combined SWE with three-dimensional ultrasound-based morphology; however, measurements were limited to discrete joint positions (25). Therefore, to address this gap, it is necessary to characterize the triceps surae muscles and the AT under the same experimental conditions using comparable measurement procedures.

Therefore, this study aimed to clarify the angle-specific stiffness characteristics of the AT and each triceps surae muscle (MG, LG, SOL) by assessing shear wave velocity—an indirect indicator of tissue material stiffness—during continuous passive ankle dorsiflexion. Using SWE, we sought to individually quantify the stiffness profiles of each tissue throughout the entire range of motion. We hypothesized that the AT would begin to stiffen earlier than the triceps surae muscles during passive dorsiflexion and would continue to show increasing shear wave velocity throughout the dorsiflexed range.

## 2 Materials and methods

### 2.1 Participants

This study was conducted at the Institute of Physical Education, Keio University (Kanagawa, Japan) from February 18 to March 19, 2025. Sample size calculations were performed using G*Power software (version 3.1.9.6; Düsseldorf, Germany). The primary outcome for the power analysis was the angle-dependent difference in shear wave velocity between the AT and the triceps surae muscles. An expected effect size of *f* = 0.25 (medium), *α* = 0.05, and powe*r* = 0.80 indicated that a minimum of 12 participants would be required to detect these differences with adequate statistical power (26). Twenty-four university track and field athletes (21 men, 3 women; age: 20 ± 1 years; height: 1.73 ± 0.07 m; weight: 60.5 ± 8.1 kg; mean ± standard deviation) participated. They were recruited from a university track and field club where they trained more than five days per week. The inclusion criterion required participants to have no current pain in the Achilles tendon. Exclusion criteria encompassed a history of AT rupture, significant ankle or foot injury/surgery, systemic diseases affecting motor function (neurological, cardiovascular, or diabetic conditions), pregnancy or lactation, and long-term use of topical or oral steroids (27). For each participant, we recorded their specialty event, last training day, medical history, dominant leg (defined as the preferred leg for kicking a ball), and the Victorian Institute of Sport Assessment–Achilles score. None of the participants was under 18 years of age. After the study's purpose, procedures, risks, and benefits were explained, written informed consent was obtained before participation. The study was approved by the Keio University Research Ethics Committee (Approval No. 24-013) and adhered to the Declaration of Helsinki and relevant ethical guidelines.

### 2.2 Experimental setup and passive dorsiflexion procedure

**Figure 1:**
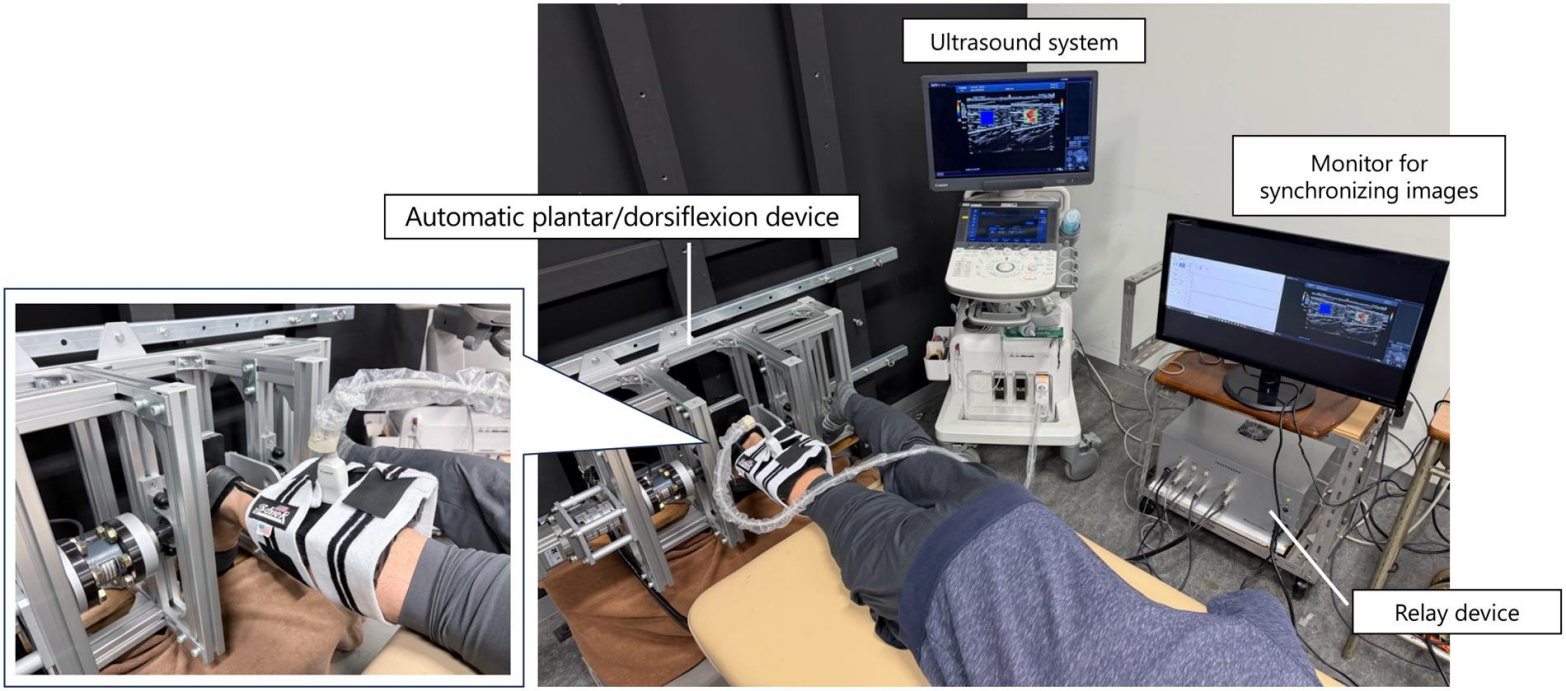
Experimental setup. Participant lying in the prone position on a dynamometer attachment, with the dominant foot secured by a belt. Ultrasound equipment was used to perform shear wave elastography (SWE) of the triceps surae and Achilles tendon (AT). For MG, LG, and SOL measurements, the ultrasound probe was fixed to the lower leg using a soft pad and elastic band to minimize movement and avoid probe pressure. For AT measurements, the probe was held manually due to interference with the dynamometer attachment. The dynamometer and ultrasound system were connected to a single display to synchronize shear wave velocity (SWV) with joint angle.

Participants were tested in a single experimental session. The experimental setup is shown in Figure 1. Each participant lay prone on a bed with the hip in a neutral position and the knee fully extended (0°). Measurements were performed on the dominant leg. Passive ankle dorsiflexion was performed using an automatic plantar/dorsiflexion device (S-18129; Takei Scientific Instruments Co., Ltd., Japan). The foot was secured to a footplate with a belt, and the rotation axis of the dynamometer was aligned with the medial and lateral malleoli. Torque and ankle joint angle data were acquired directly from the dynamometer's output system, which includes an integrated analog-to-digital converter for real-time angle measurement. These signals were recorded at a sampling frequency of 1,000 Hz and collected on a PC equipped with a relay device (AIO AI-1608AY-USB; Takei Scientific Instruments Co., Ltd., Japan) using dedicated software [Ver. 1.1.0 (S-19040)]. First, the maximum passive dorsiflexion angle was determined. The ankle was passively dorsiflexed at 1° /s, and participants pressed a handheld switch when they reached their maximum tolerable stretch in the posterior lower leg. This procedure ensured that the measurement reflected a purely passive stretch, without voluntary or reflexive muscle activation. An angle corresponding to 80% of the individual maximum dorsiflexion angle was then calculated, as electromyographic activity of the plantar flexors is known to increase beyond this range during passive dorsiflexion (28). During the passive dorsiflexion movement, shear wave velocity (SWV) of the MG, LG, SOL, and AT was measured using SWE. The primary outcome was the percentage change in SWV at each joint angle.

### 2.3 Shear wave elastography measurement

An ultrasound system with SWE capability (Aplio a Verifia, Canon Medical Systems Corporation, Japan) and a linear probe (65 mm, 8–14 MHz) were used to acquire B-mode ultrasound images in the longitudinal direction with color mapping of SWV values for the triceps surae and AT during passive dorsiflexion. All measurements were performed by a licensed physical therapist with 13 years of clinical experience and extensive expertise in musculoskeletal anatomy and ultrasound imaging, ensuring consistent data acquisition. The probe was positioned along the longitudinal axis of the muscle or tendon and placed perpendicular to the skin. Alignment was confirmed by ensuring that both the superficial and deep aponeuroses were visible in the B-mode image. For muscle measurements, probe orientation was further adjusted to maximize fascicle visibility on B-mode imaging, aligning the imaging plane as parallel as possible to the dominant fiber direction to mitigate anisotropy-related bias in SWE measurements (29). Probe pressure was minimized, and the device's smoothing level was set to an intermediate spatial level (3/5). To standardize probe positioning, all skin markings were made with the lower leg in the resting position at the edge of the bed, ensuring consistent anatomical alignment across participants. Measurement sites were marked as follows: the maximal bulge of the MG and LG was identified at approximately 30% of the proximal lower leg length; the SOL was identified distal to the myotendinous junction of the LG (17); and the AT was identified at the site of greatest tendon thickness, approximately 2 cm from its insertion. Probe placement locations were marked on the skin with a pen. For the MG, LG, and SOL, the probe was mounted on a custom-made soft pad and secured to the lower leg using an elastic band to prevent movement and ensure consistent placement without applying pressure. In contrast, the AT could not be measured using this fixation method due to interference with the dynamometer attachment. Therefore, the operator held the probe by hand with minimal contact to avoid compression. A gel pad was placed on the concave surface of the tendon, and the probe was positioned on top of it during AT measurements. Proper alignment was verified by confirming clear visualization of both superficial and deep aponeuroses and stable shear-wave propagation.

**Figure 2:**
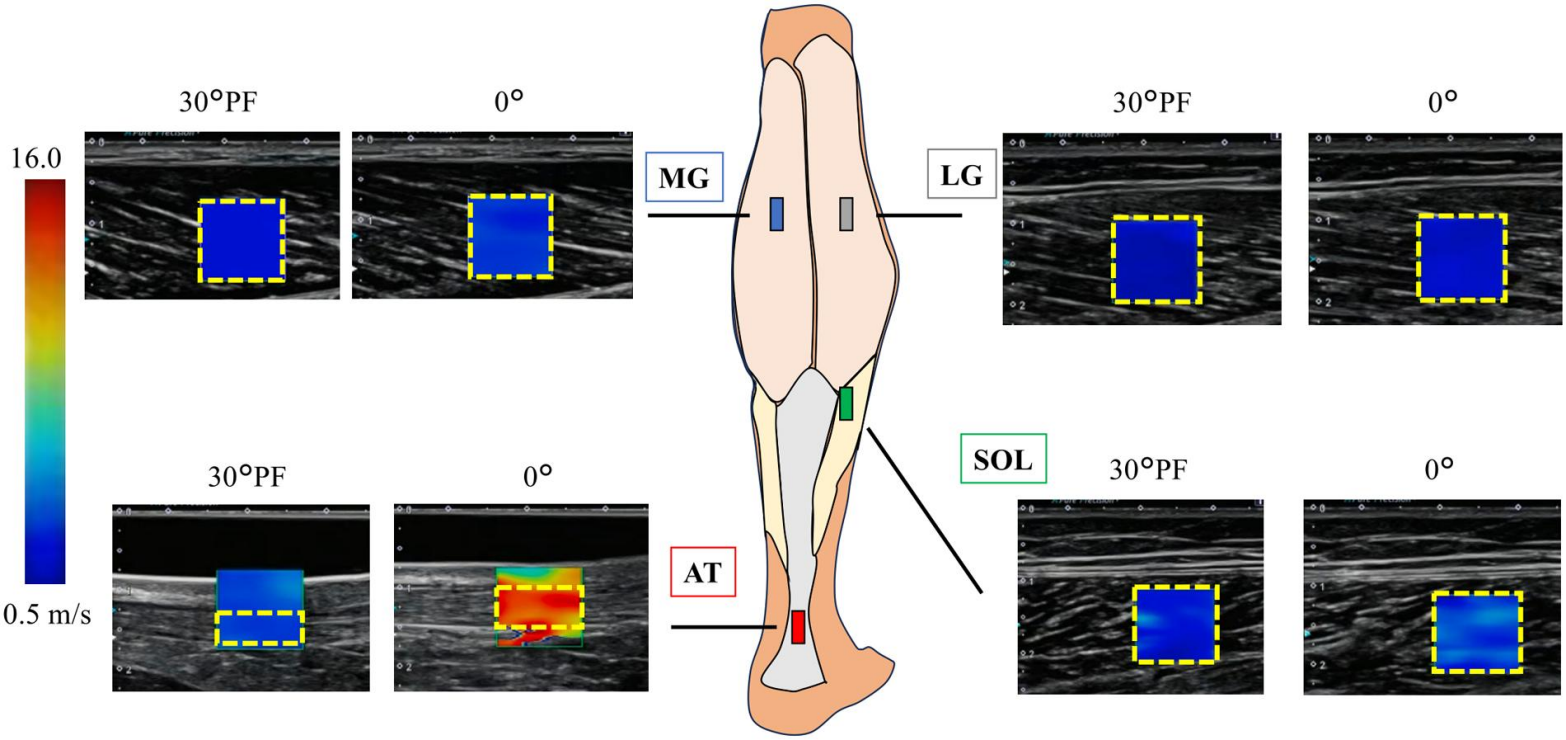
Anatomical landmarks for ultrasonography and shear wave elastography. Representative B-mode images with shear wave velocity (SWV) color mapping for the triceps surae muscles and the Achilles tendon (AT) obtained at 30° plantarflexion (PF) and 0° (neutral; maximal dorsiflexion achievable for AT measurements). A color bar (0.5–16.0 m/s) is included to indicate the shear wave velocity (SWV) scale. The analysis region of interest (ROI) is shown with a yellow dashed outline in each image, and depth markers are displayed on the left side of each panel. Images were acquired with the probe aligned parallel to the muscle or tendon fascicles. MG, medial gastrocnemius; LG, lateral gastrocnemius; SOL, soleus; AT, Achilles tendon.

Figure 2 shows the marked sites and a B-mode ultrasound image with SWV color mapping at the neutral (0°) ankle position. To account for muscle conditioning, five cycles of passive ankle movement were performed at 5° /s from 30° PF, where passive torque is considered zero (30), to 80% of the maximum dorsiflexion angle (31). SWE measurements were then taken at each site (MG, LG, SOL, and AT) during passive dorsiflexion at 1° /s, with a 1-minute rest period between measurements at each site. Finally, to check for any stretching effect from repeated measurements, SWE of the MG was repeated. Each site was measured three times, and one representative trial was used for analysis. Intraclass correlation coefficients [ICC (1, 3), absolute agreement] were calculated between the three repeated measurements and the subsequent single measurement in six participants to evaluate the reliability of using a single representative measurement. The ICC values were high across all sites (ICC = 0.85–0.99), indicating excellent measurement reliability. The total experimental duration, including preparation and data collection, was approximately 90 min per participant.

### 2.4 Data processing

SWV data were processed using the analysis software built into the ultrasound system. For each measurement, a region of interest (ROI) was defined: a 1 cm × 1 cm square for each muscle, and for the AT, a 1 cm wide region with maximal height to encompass the tendon substance. Areas with visible shear wave disturbances (artifacts) were avoided, and analysis was limited to regions exhibiting uniform wave propagation.

To synchronize the SWE data with joint kinematics, the real-time dynamometer outputs and continuously acquired SWE images (recorded at one frame per second during the 1°/s passive movement) were displayed simultaneously using the Picture-in-Picture function. This enabled the extraction of angle-matched SWV values from sequential frames.

Slack angle for each structure (MG, LG, SOL, and AT) was determined from the ankle angle–SWV relationship as the ankle angle at which SWV rose above baseline variation, following previous SWE studies (15). Specifically, baseline variation was quantified from the SWV plateau observed in the plantarflexed range, and the slack angle was defined as the first ankle angle at which SWV exceeded this baseline and showed a consistent upward trend with further dorsiflexion.

SWV values were expressed as the percentage increase from the baseline measured at 30° PF. SWV was acquired continuously; however, values were extracted at 5° increments and summarized as mean ± standard deviation to match the inferential statistics and reduce frame-to-frame variability. The analytic range for the AT was set from 30° PF to 0° because partial saturation of the ultrasound system (upper Young's modulus limit: 700 kPa; ≈15.3 m/s) occurred as dorsiflexion progressed. The neutral position (0°) represented the last angle at which valid tendon data were consistently obtained for all participants. In contrast, triceps surae muscles (MG, LG, and SOL) remained within the measurable range throughout dorsiflexion. However, for comparative purposes, muscle data were analyzed only up to 0° to match the AT, for which valid measurements were consistently obtained only up to the neutral position.

### 2.5 Statistical analysis

All data are presented as mean ± standard deviation. Statistical analyses were performed using IBM SPSS Statistics for Mac (v30.0; Armonk, NY, USA). To examine the presence of a stretching effect (the influence of repeated passive dorsiflexion on stiffness), the difference in SWV at 80% of the maximum dorsiflexion angle between the first (MG-1st) and second (MG-2nd) MG measurements was assessed using a paired t-test after verifying normality with the Shapiro–Wilk test. Subsequently, a two-way repeated-measures analysis of variance (ANOVA) was conducted to compare SWV values across sites and angles. The within-subject factors were angle (seven levels: 30° PF, 25° PF, 20° PF, 15° PF, 10° PF, 5° PF, 0°) and measurement site (four levels: MG, LG, SOL, AT). This analysis was performed using the calculated percentage change in SWV data. If an interaction or main effect was observed, *post hoc* comparisons were conducted using the Bonferroni correction. In addition, slack angle was compared across measurement sites (MG, LG, SOL, and AT) using a one-way repeated-measures ANOVA with Greenhouse–Geisser correction applied when sphericity was violated, followed by Bonferroni-adjusted pairwise comparisons. Normality of residuals for all stiffness measurements (MG, LG, SOL, and AT) was assessed using the Shapiro–Wilk test and by visual inspection of Q–Q plots. Some conditions showed minor deviations from normality; however, no significant departures were observed, and repeated-measures ANOVA is generally robust to moderate non-normality. Statistical significance was set at *p* < 0.05.

## 3 Results

The physical characteristics of the 24 participants are summarized in Table 1. Regarding the effect of repeated passive dorsiflexion on stiffness, no significant difference was found between the MG-1st and MG-2nd measurements (5.78 ± 0.49 m/s vs. 5.69 ± 0.63 m/s, *p* = 0.45).

**Table 1:** Characteristics of the study participants.

Characteristics	*N* = 24	Males (*n* = 21)	Females (*n* = 3)
Age, mean (SD)	20.2 (1.3)	20.1 (1.2)	21.0 (1.0)
Height, mean (SD)	172.6 (6.9)	174.9 (4.4)	157.8 (3.0)
Weight, mean (SD)	61.1 (8.3)	62.8 (6.2)	49.8 (2.8)
Speciality event, *n* (%)			
Sprinter	8 (33.3)	7 (29.1)	1 (4.2)
Middle/Long distance runner	13 (54.2)	11 (45.8)	2 (8.3)
Jumper	2 (8.3)	2 (8.3)	-
Combined	1 (4.2)	1 (4.2)	-
Leg dominance, *n* (%)			
Right	23 (95.8)	20 (83.3)	3 (12.5)
Left	1 (4.2)	1 (4.2)	-
VISA-A score, mean (SD)	100.0 (-)	-	-
Tendon thickness (mm), mean (SD)	4.8 (0.3)	4.9 (0.3)	4.7 (0.4)
Maximum dorsiflexion angle (°)	27.2 (4.9)	26.5 (4.4)	32.1 (6.5)

VISA-A Score, victorian institute of sport assessment–achilles.

**Figure 3:**
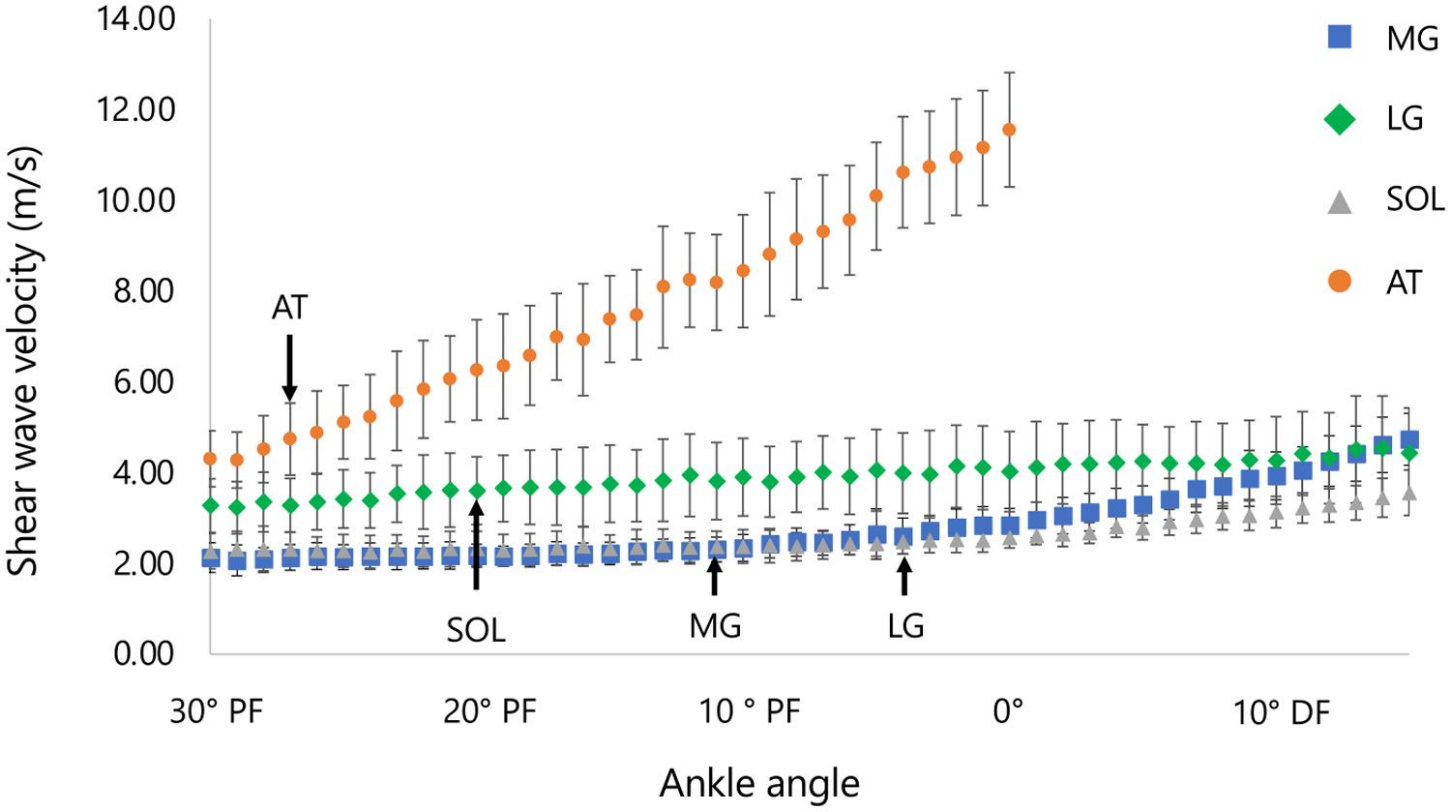
Shear wave velocity–angle relationships during passive dorsiflexion. MG, medial gastrocnemius; LG, lateral gastrocnemius; SOL, soleus; AT, Achilles tendon. Muscle stiffness (MG, LG, SOL) was measurable across the full dorsiflexed range, including beyond 0°, whereas AT stiffness reached the upper measurement limit of the ultrasound device at 0°, and values beyond 0° were therefore not analyzed. Arrows in shear modulus-joint angle curves indicate the slack angles of MG, LG, Sol, and AT. Data represent mean ± SD.

The SWV and shear modulus at each ankle joint angle for each measurement site (MG, LG, SOL, and AT) are presented in Table 2; Figure 3. A significant interaction was observed between measurement site and ankle angle for the percentage change in SWV during passive dorsiflexion (*p* < 0.05). A significant simple main effect of ankle angle was also found for each measurement site (MG: F = 18.523, *p* < 0.05; LG: F = 3.801, *p* < 0.05; SOL: F = 10.330, *p* < 0.05; AT: F = 70.109, *p* < 0.05). Slack angles were identified at 11.7 ± 5.2° PF for the MG, 4.2 ± 7.3° PF for the LG, 20.1 ± 4.4° PF for the SOL, and 27.4 ± 1.7° PF for the AT (Figure 3). Slack angle differed significantly across sites [F(2.293, 52.728) = 101.657, *p* < 0.05, *η*p^2^ = 0.815], and Bonferroni-adjusted *post hoc* tests showed significant differences between all sites (LG > MG > SOL > AT; all *p* ≤ 0.05).

**Table 2:** Shear wave velocity and shear modulus at each ankle joint angle.

Parameter	Region	Ankle Angle									
		30° PF	25° PF	20° PF	15° PF	10° PF	5° PF	0	5° DF	10° DF	15° DF
SWV (m/s)	MG	2.13 ± 0.33	2.14 ± 0.27	2.17 ± 0.25	2.22 ± 0.25	2.35 ± 0.29	2.65 ± 0.55	2.84 ± 0.37	3.30 ± 0.41	3.93 ± 0.53	4.74 ± 0.57
	LG	2.27 ± 0.39	2.28 ± 0.36	2.33 ± 0.38	2.32 ± 0.31	2.38 ± 0.37	2.44 ± 0.28	2.57 ± 0.23	2.79 ± 0.27	3.13 ± 0.34	3.56 ± 0.50
	SOL	3.27 ± 0.59	3.42 ± 0.64	3.61 ± 0.74	3.76 ± 0.85	3.90 ± 0.80	4.05 ± 0.90	4.02 ± 0.88	4.25 ± 0.81	4.27 ± 0.96	4.43 ± 1.00
	AT	4.31 ± 0.62	5.11 ± 0.81	6.26 ± 1.11	7.39 ± 0.95	8.44 ± 1.24	10.09 ± 1.19	11.55 ± 1.26			
Shear modulus (kPa)	MG	4.53 ± 1.66	4.61 ± 1.31	4.76 ± 1.25	4.95 ± 1.15	5.56 ± 1.56	7.39 ± 4.56	8.21 ± 2.31	11.06 ± 2.89	15.82 ± 4.30	23.03 ± 5.72
	LG	5.15 ± 2.15	5.41 ± 1.96	5.71 ± 2.23	5.55 ± 1.60	5.87 ± 2.20	6.17 ± 1.83	6.75 ± 1.37	7.92 ± 1.64	10.06 ± 2.32	13.08 ± 3.87
	SOL	11.24 ± 4.17	12.37 ± 4.94	13.79 ± 6.23	15.25 ± 7.87	16.45 ± 8.34	17.74 ± 8.67	17.53 ± 8.34	19.32 ± 7.44	20.14 ± 9.95	21.47 ± 10.75
	AT	19.10 ± 5.87	27.03 ± 9.10	41.24 ± 15.31	56.39 ± 15.11	74.22 ± 21.00	105.75 ± 25.39	137.34 ± 26.88			

Values are means ± standard deviation. MG, medial gastrocnemius; LG, lateral gastrocnemius; SOL, soleus; SWV, shear wave velocity; PF, planter flexion; DF dorsiflexion. Ankle angles are defined such that negative values indicated plantar flexion and positive values indicated dorsiflexion.

**Figure 4:**
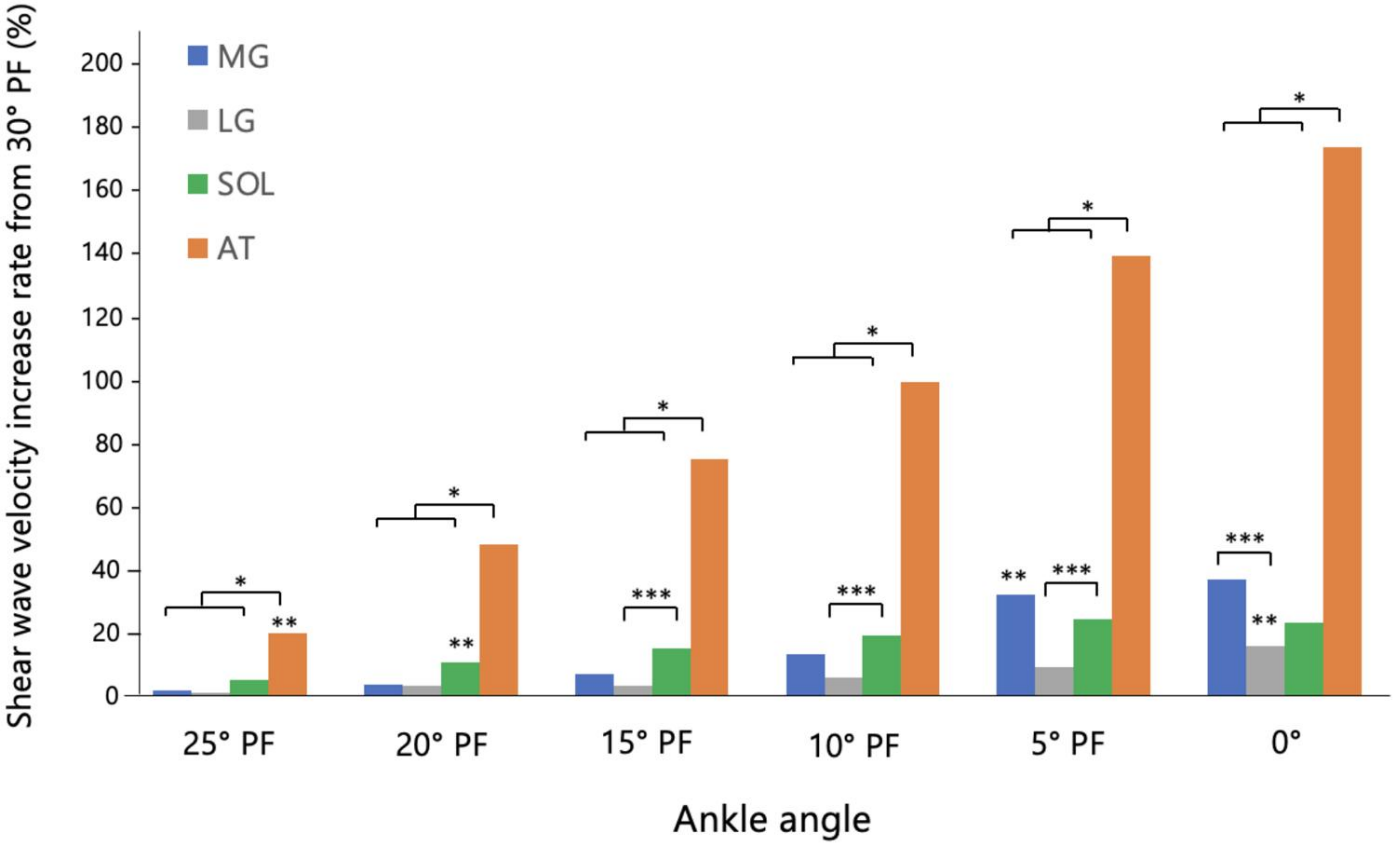
Histogram of the rate of increase relative to 30° PF. Results of repeated-measures ANOVA (*p* < 0.05) followed by Bonferroni *post hoc* test. *: Significant difference between the AT and all three muscles. **: The point at which the rate of change significantly increased for each measurement site (MG, LG, SOL, and AT). ***: Significant differences between muscle groups at each angle. MG, medial gastrocnemius; LG, lateral gastrocnemius; SOL, soleus; AT, Achilles tendon; PF, planter flexion.

Figure 4 illustrates the percentage change in SWV from the baseline value at 30° PF. The percentage change in SWV for the AT was significantly higher than its baseline value at 30° PF for all joint angles from 25° PF to 0°. For the triceps surae muscles, the percentage change in SWV was significantly higher than baseline from 10° PF to 0° for the MG, at 0° for the LG, and from 20° PF to 0° for the SOL. Furthermore, the percentage change in SWV for the AT was significantly greater than that of all three triceps surae muscles at every joint angle from 25° PF onward. Among the triceps surae muscles, the SOL showed a significantly greater change than the LG at 15° PF, 10° PF, and 5° PF, while the MG exhibited a significantly greater change than the LG at 0°.

## 4 Discussion

In this study, we comprehensively assessed the stiffness of individual triceps surae muscles and the AT in healthy adult university track and field athletes within the same session. The ankle was passively moved at a constant speed from 30° PF to 80% of the maximum dorsiflexion angle. Our results showed that the AT exhibited an immediate and significant increase in stiffness from the early phase of passive ankle movement, beginning in the plantar-flexed range and progressively continuing toward dorsiflexion. In contrast, the MG, LG, and SOL showed little initial change, with stiffening beginning at significantly different angles than the AT. The range of motion at which each muscle began to increase in stiffness also varied. Among the triceps surae, the SOL was the first to show a significant increase in stiffness, followed by the MG and LG. These findings support our study hypothesis that the AT would exhibit an earlier and more pronounced increase in stiffness than in the triceps surae muscles during passive dorsiflexion.

The AT stiffness (SWV) measured at the 0° ankle angle was 11.55 ± 1.26 m/s. Previous studies have reported AT stiffness values at 0° of 7.5 m/s (32), 9.04 m/s (33), and 15.5 m/s (16), validating the plausibility of our results. Other studies have shown that student-athletes generally exhibit higher stiffness than non-athletes (34); however, since our values fall within the previously reported range, it is unlikely that our participants were atypically biased. Regarding the change in AT stiffness during dorsiflexion, a significant increase was observed as early as 5° from the starting position of 30° PF, with stiffness continuing to increase linearly up to 0°. This supports previous findings that AT stiffness increases immediately with the onset of dorsiflexion from a plantar-flexed position (20). The behavior can be explained by the relationship between AT angle and its slack length. Previous studies (15, 20) have shown that the slack length of the AT is reached at a greater plantar-flexion angle (approximately 43° PF) than that of the MG (approximately 18° PF to 25° PF). Since our protocol began at 30° PF due to equipment limitations, it is possible that the tendon had already exceeded its slack length and therefore immediately resisted further stretching. Moreover, in the range beyond 20° PF, which was not investigated in the previous study by Hug et al. (20), Slane and Thelen (35, 36) reported that the AT elongates by approximately 5–10 mm between 30° PF and 0°. This supports the likelihood that the AT continues to elongate beyond its slack length, leading to the linear increase in stiffness observed in our study. From a biomechanical perspective, according to the Hill-type muscle model, muscle tissue functions as the contractile element (CE), whereas tendon tissue serves as the passive element (PE) (37). The linear increase in AT stiffness after reaching its slack length is therefore characteristic of tendon tissue, which is primarily composed of robust collagen fibers, unlike muscle.

In contrast to the AT, the triceps surae muscles exhibited a delayed and curvilinear increase in stiffness during passive dorsiflexion, remaining low in the plantar-flexed range before rising at higher angles. A curvilinear (nonlinear) increase in passive triceps surae muscle stiffness with dorsiflexion is expected based on prior SWE studies (14, 17). Consistent with our slack-angle analysis, muscle-specific slack behavior differed across triceps surae muscles and the AT. Intermuscle differences in slack behavior within the triceps surae have been reported previously, and distinct slack behavior between gastrocnemius and the AT has also been reported (20). This indicates that the triceps surae contribute less to passive resistance in the plantar-flexed range compared with the AT. Among the triceps surae, the SOL was the first to show a significant increase in stiffness. In line with this pattern, our slack-angle analysis also indicated that the SOL exhibited the earliest slack behavior among the triceps surae muscles. All ultrasound measurements were performed by the same operator, with the transducer aligned parallel to the fascicle direction at each site to ensure consistency across muscles. Minor anatomical variations in fascicle orientation were carefully adjusted during probe positioning to minimize measurement bias. A previous anatomical study reported that, in addition to the tendons of the MG, LG, and SOL forming the AT with a twisted structure, the SOL tendon lies deep within the AT near its core (38). Slane and Thelen (35, 36) also observed greater displacement in this same region during passive stretch. These findings suggest that AT fibers continuous with the SOL are stretched earlier, consistent with our observation that SOL stiffness increased from 20° PF. The delayed changes in MG and LG stiffness indicate that their contribution to passive resistance becomes more prominent as the ankle approaches the neutral position. The earlier involvement of the SOL in our study compared with previous reports may be attributed to the participant positioning. Prior research has shown that muscle cross-sectional area varies between sitting and supine positions due to gravity (39), suggesting that our prone setup might have influenced the initial tension and subsequent stiffening profile of the SOL. Methodological differences may also explain the discrepancy. Zimmer et al. also used a prone setup; however, they measured the SOL at a more proximal, deep region and obtained static SWE measurements, whereas we assessed a distal site and acquired data continuously during passive dorsiflexion (24). These differences in probe location and measurement mode likely affected local tissue strain, contributing to the earlier stiffening onset observed in our study. This interpretation is supported by evidence that muscle mechanical behavior can be spatially heterogeneous, with region-specific differences reported even within the same muscle (40). Moreover, proximodistal heterogeneity in MG fascicle–aponeurosis dynamics and strain during passive ankle rotation has been reported (41), suggesting that more distal measurement sites, closer to the ankle joint, may capture different local strain states and, consequently, different stiffness–angle profiles.

The primary finding of this study is that the AT significantly contributes to the stiffness of the triceps surae–tendon unit, particularly in the plantar-flexed range. Achilles tendinopathy is one of the most common running-related injuries (7). A forefoot strike pattern, characterized by plantar flexion at ground contact, has been identified as a risk factor for Achilles tendinopathy (42). In this pattern, ground contact occurs during plantar flexion (43), and the AT experiences higher loading from early to mid-stance compared with a rearfoot strike pattern (44). This suggests that the tendon bears a greater mechanical burden, as it contributes more to the stiffness of the muscle-tendon unit than the muscles during ground contact in plantar flexion. Therefore, AT stiffness in the plantar-flexed range may be an important factor in the development of tendinopathy. Previous studies have reported that patients with Achilles tendinopathy exhibit lower AT stiffness compared with healthy controls, although these assessments were limited to resting (45) or neutral (0°) positions (46). Future studies should examine whether reduced AT stiffness in the plantar-flexed range is also associated with AT injury. It should be noted that these findings are based on passive mechanical properties. During dynamic activities such as running, muscle activation significantly increases muscle stiffness, which may alter the stiffness ratio observed in our passive measurements. In addition, these findings may be clinically relevant for rehabilitation after AT rupture repair, during which ankle position is typically progressed gradually from plantarflexion toward neutral dorsiflexion. Tendon elongation is a well-recognized concern after rupture repair and can continue for several months, potentially influencing functional recovery (47, 48). Because immobilization angle and ankle position affect tendon loading and elongation, our observation that the tendon contributes significantly to passive stiffness in plantarflexion suggests that careful control of ankle angle and loading progression may be warranted even in relatively plantarflexed postures during early rehabilitation.

A key limitation of this study is that the starting angle of passive dorsiflexion (30° PF) was restricted by the available equipment. Previous studies measuring AT stiffness used ranges up to 50° PF (20), whereas those focused solely on muscles measured from 40° PF (14, 15). In our study, measurements below 30° PF were not feasible because participants experienced discomfort during fixation of the ankle to the dynamometer. Furthermore, for the AT, SWE measurements approached the device's upper output limit for Young's modulus (700 kPa), resulting in partial saturation in the dorsiflexed range and restricting analyzable data to the neutral (0°) position. This limitation did not apply to the triceps surae muscles, which remained within the measurable range; therefore, future studies should evaluate the entire triceps surae–AT complex across the full range of motion. In addition, probe positioning was standardized using skin markings at rest; however, fascicle elongation during dorsiflexion inevitably causes muscle tissue to shift relative to the skin surface. For example, MG fascicles lengthen from ∼50 mm to ∼80 mm (49), indicating that some probe-to-tissue displacement is inherent to SWE measurements under dynamic joint motion. This constraint is consistent with previous studies using similar protocols (17, 20) but should be considered when interpreting the measurements. Moreover, we examined the muscle–tendon unit under passive conditions. In contrast, during running, the triceps surae are activated prior to and throughout ground contact, significantly increasing muscle stiffness and potentially reducing the stiffness imbalance observed in passive states. Therefore, the present findings should be interpreted as fundamental mechanical characteristics rather than direct representations of dynamic loading during running.

In conclusion, this study demonstrated that the AT and triceps surae exhibit distinct stiffness change patterns during passive dorsiflexion. The tendon's stiffness increases significantly from a more plantar-flexed position than the muscles, suggesting that the AT predominantly contributes to the initial resistive behavior of the triceps surae–tendon unit. However, this interpretation should be made cautiously, as connective tissues surrounding the joint and the joint capsule may also contribute to passive resistance in the plantar-flexed range. From a practical standpoint, these findings highlight the importance of considering tendon stiffness when managing loading conditions and rehabilitation in athletes. For example, gradual loading programs that account for tendon mechanical properties during plantar-flexed postures may help optimize recovery and reduce the risk of tendinopathy. By being the first to continuously evaluate the stiffness of both muscle and tendon under identical experimental conditions, this study offers important new insights into tendon health, function, and injury prevention.

## Data Availability

The raw data supporting the conclusions of this article will be made available by the authors, without undue reservation.
